# Lower BMI in Tanzanian Adults with *Schistosoma mansoni* Infection is Not Explained by Differences in Serum Adipocytokine Levels

**DOI:** 10.5334/gh.1436

**Published:** 2025-06-05

**Authors:** Khanh Pham, Enock Miyaye, Maureen Ward, Danielle de Jong, Govert J. van Dam, Paul L. A. M. Corstjens, Jennifer A. Downs, Robert N. Peck, Hyasinta Jaka

**Affiliations:** 1 Division of Infectious Diseases, Weill Cornell Medicine, NY, USA; 2 Center for Global Health, Weill Cornell Medicine, NY, USA; 3 Department of Medicine, Weill Bugando School of Medicine, Mwanza, Tanzania; 4 Department of Cell and Chemical Biology, Leiden University Medical Center, Leiden, The Netherlands; 5 Department of Parasitology, Leiden University Medical Center, Leiden, The Netherlands; 6 Mwanza Intervention Trials Unit/National Institute for Medical Research, Mwanza, Tanzania; 7 Department of Internal Medicine, Catholic University of Health and Allied Sciences-Bugando, Mwanza, Tanzania

**Keywords:** *Schistosoma mansoni*, adipocytokines, body mass index, Cardiometabolic diseases, pathways, Tanzanian adults

In 2022, the World Health Organization reported that 1 in 8 people globally were living with obesity, which is defined as a body mass index (BMI) ≥30 kg/m² (1). Adipocytokines are immunomodulatory proteins secreted by adipocytes that act in feedback loops linking obesity and cardiometabolic disease, which typically include leptin, adiponectin, interleukin (IL)-6, IL-18, and tumor necrosis factor-alpha (TNF-a) (2). Several other cytokines, including IL-10 and interferon-gamma (IFN-γ), also play key roles in inflammation and cardiometabolic disease (2). Together, these adipocytokines are crucial for regulating hunger, body metabolism, endocrine function, and immune responses. Typically, higher leptin levels and lower adiponectin levels are associated with greater fat mass (2).

Infection with *Schistosoma mansoni* is associated with lower BMI, less obesity, and less cardiometabolic disease, although the mechanism that underlies this effect remains unclear (3,4). Some studies have hypothesized that *S. mansoni* infection may affect adipokine- or cytokine-related pathways, thus blocking both obesity and obesity-associated cardiometabolic disease (5).

To investigate potential immunometabolic pathways between schistosome infection and BMI, we employed targeted enzyme-linked immunosorbent assays (ELISAs) to quantify circulating adipocytokines that play a central role in pathogenesis related to obesity and cardiometabolic disease (2). Serum levels of leptin, adiponectin, IL-6, TNF-α, IL-10, IL-18, and IFN-γ were therefore measured in 144 adults aged 18–50 years residing in northwest Tanzania, in an area highly endemic for *S. mansoni*, between July 2022 and April 2023. *S. mansoni* infection was defined as a serum circulating anodic antigen (CAA) level ≥30 pg/mL, the presence of at least one egg with *S. mansoni* morphology in stool, and the absence of *Schistosoma haematobium* eggs in urine (6,7). Individuals without infection had a serum CAA < 30 pg/mL and were negative for *S. mansoni* in stool and for *S. haematobium* in urine (6,7). Screening and enrollment followed previously published procedures (6), and data on diet, exercise, and BMI were collected using standardized, translated, and validated questions from the World Health Organization’s Stepwise Approach to Surveillance (STEPS) for the risk factors of cardiovascular disease. Blood samples were collected to measure serum adipocytokine levels using the Luminex Magpix® Multiplexing System (Austin, TX, USA). Samples were run in singlet and diluted to 1:100 for the adiponectin assay and undiluted for the other adipocytokine assays, as recommended by the manufacturer (ThermoFisher Scientific; Waltham, MA, USA). Every plate run contained standard curve samples run in duplicate. Samples out of the standard range were repeated at a higher or lower dilution.

Our primary outcomes were achieved by measuring serum adipocytokine levels. We performed linear regression analyses to determine the association between schistosome infection (our primary exposure of interest) and adipocytokine levels. We adjusted for age and sex to account for baseline demographic differences between the schistosome-infected and uninfected groups. We further analyzed the association between BMI and adipocytokine levels using the same methods.

In our study cohort, most sociodemographic characteristics were similar between 61 adults (42.4%) with *S. mansoni* infection and 83 adults (57.6%) without infection (Supplemental Table 1). However, uninfected individuals tended to be older on average (mean age 34.5 years vs. 31.6 years, p = 0.07) and had a higher proportion of women (51.8% vs. 36.1%, p = 0.06).

**Supplemental Table 1:** Baseline demographic characteristics and outcome measurements of 144 adults with and without *Schistosoma mansoni* infection.


DEMOGRAPHIC CHARACTERISTICS		

CHARACTERISTIC n (%) OR MEAN (SD)	SCHISTOSOME-INFECTED (n = 61)	SCHISTOSOME-UNINFECTED (n = 83)

Age (years)	31.6 (8.4)	34.5 (10.0)

Sex (female)	22 (36.1%)	43 (51.8%)

Marital status		

Single	12 (19.7%)	15 (18.1%)

Married	40 (65.6%)	50 (60.2%)

Other	9 (14.8%)	18 (21.7%)

Years attended school	7.6 (2.6)	8.2 (2.9)

Past schistosome treatment	16 (26.2%)	17 (20.5%)

Vigorous exercise per week (min)	4082 (1000)	4122 (1161)

Active tobacco use	12 (19.7%)	9 (10.8%)

Active alcohol use	12 (19.7%)	17 (20.5%)

**OUTCOME MEASUREMENTS**		

**CHARACTERISTIC n (%) OR MEAN (SD; RANGE)**	**SCHISTOSOME-INFECTED (n = 61)**	**SCHISTOSOME-UNINFECTED (n = 83)**

Body mass index (kg/m^2^)	21.7 (4.5; 15.5–44.1)	23.7 (5.6; 15.5–44.6)

Body mass index categories		

<18.5	9 (14.8%)	11 (13.3%)

≥18.5 and <25	44 (72.1%)	44 (53.0%)

≥25 and <30	4 (6.6%)	19 (22.9%)

≥30	4 (6.6%)	9 (10.8%)

Leptin (µg/mL)	3.9 (6.4; 0.04–29.3)	2.9 (4.7; 0.01–26.3)

Adiponectin (µg/mL)	15.8 (19.2; 0.4–114.1)	15.8 (16.0; 0.6–74.4)

Adiponectin-to-leptin ratio	56.6 (128.2; 0.3–737.5)	94.4 (303.1; 0.2–2394.1)

IL-6 (pg/mL)	3.7 (1.2; 2.0–9.0)	4.2 (3.7; 1.0–33.0)

TNF-α (pg/mL)	5.3 (0.9; 4.0–8.0)	6.2 (8.0; 3.0–78.0)

IL-10 (pg/mL)	7.0 (3.7; 3.0–22.0)	7.6 (6.3; 2.0–44.5)

IFN-γ (pg/mL)	4.8 (4.5; 0–26.6)	7.8 (10.6; 0–89.8)

IL-18 (pg/mL)	53.2 (38.9; 2.7–227.4)	68.5 (50.0; 2.7–280.0)


IL, interleukin; IFN-γ, interferon-gamma; TNF-α, tumor necrosis factor-alpha.

Individuals with schistosome infection had a lower BMI compared to those without infection (mean BMI of 21.7 vs. 23.7 kg/m², p = 0.026), a finding consistent with our previous research (4). Furthermore, a much lower proportion of individuals with *S. mansoni* infection were classified as overweight (BMI ≥ 25 and < 30 kg/m²) or obese (BMI ≥ 30 kg/m²) compared to those without infection (13.1% vs. 33.7%, p = 0.035).

Regarding serum adipocytokines, individuals with *S. mansoni* infection had lower IFN-γ (mean 4.8 vs. 7.8 pg/mL, p = 0.048) and IL-18 (mean 53.2 vs. 68.5 pg/mL, p = 0.032) levels than their uninfected counterparts after adjusting for age and sex (Table 1). No differences were observed in serum leptin, adiponectin, IL-6, TNF-α, or IL-10 level, or in the adiponectin-to-leptin ratio. Additionally, no significant associations existed between the adipocytokines and BMI (Supplemental Table 2).

**Table 1:** Association between adipocytokines and *Schistosoma mansoni* Infection status in 144 adults.


ADIPOCYTOKINE	UNADJUSTED (COEFFICIENT (95% CI), p-VALUE)	ADJUSTED FOR AGE AND SEX (COEFFICIENT (95% CI), p-VALUE)

Leptin	1.0 (–0.8, 2.9), p = 0.26	1.3 (–0.6, 3.2), p = 0.17

Adiponectin	0.06 (–5.8, 5.9), p = 0.98	–0.4 (–6.5, 5.6), p = 0.88

Adiponectin-to-leptin ratio	–37.8 (–119.7, 44.1), p = 0.36	–41.5 (–125.1, 42.2), p = 0.33

IL-6	–0.5 (–1.5, 0.5), p = 0.30	–0.4 (–1.4, 0.6), p = 0.46

TNF-α	–0.8 (–2.9, 1.2), p = 0.42	–0.6 (–2.7, 1.5), p = 0.57

IL-10	–0.6 (–2.4, 1.2), p = 0.54	–0.6 (–2.4, 1.3), p = 0.55

IFN-γ	–2.9 (–5.8, –0.1), p = 0.044	–3.0 (–5.9, –0.03), p = 0.048

IL-18	–15.3 (–30.5, –0.1), p = 0.049	–17.1 (–32.8, –1.5), p = 0.032


CI, confidence interval; IL, interleukin; IFN-γ, interferon-gamma; TNF-α, tumor necrosis factor-alpha.

**Supplemental Table 2:** Association between adipocytokines and BMI in 144 adults.


ADIPOCYTOKINE	UNADJUSTED (COEFFICIENT (95% CI), p-VALUE)	ADJUSTED FOR AGE AND SEX (COEFFICIENT (95% CI), p-VALUE)

Leptin	0.1 (–0.1, 0.3), p = 0.19	0.1 (–0.1, 0.2), p = 0.25

Adiponectin	–0.02 (–0.07, 0.03), p = 0.43	–0.01 (–0.06, 0.04), p = 0.64

Adiponectin-to-leptin ratio	–0.0003 (–0.004, 0.003), p = 0.86	0.0002 (–0.003, 0.004), p = 0.92

IL-6	0.1 (–0.2, 0.4), p = 0.33	0.08 (–0.2, 0.4), p = 0.57

TNF-α	0.06 (–0.08, 0.2), p = 0.38	0.04 (–0.1, 0.2), p = 0.53

IL-10	–0.03 (–0.2, 0.1), p = 0.76	–0.02 (–0.2, 0.1), p = 0.78

IFN-γ	0.008 (–0.1, 0.1), p = 0.88	0.005 (–0.1, 0.1), p = 0.92

IL-18	0.005 (–0.01, 0.02), p = 0.57	0.007 (–0.01, 0.03), p = 0.43


BMI, body mass index; IL, interleukin; IFN-γ, interferon-gamma; TNF-α, tumor necrosis factor-alpha.

To the best of our knowledge, these are the first cross-sectional data to explore the relationship between *S. mansoni* infection, BMI, and serum adipocytokines. Consistent with prior human and animal studies (3,4,8), our data again demonstrate that *S. mansoni* infection may be associated with reduced fat mass, as indicated by lower BMI. On the other hand, we observed only minor differences between schistosome infection and serum adipocytokines—particularly the levels of IFN-γ and IL-18—and none of the adipocytokines was associated with BMI in the Tanzanian population.

The underlying mechanism for these observed adiposity differences remains unclear. Previous studies have suggested that *Schistosoma* worms may activate immune cell populations, such as anti-inflammatory macrophages, which influence host metabolism and contribute to metabolic diseases (3,4). In cardiovascular disease research from high-income countries, individuals with obesity typically have lower adiponectin and higher leptin levels (2). Limited data exist from sub-Saharan Africa. In our study, we did not observe any differences in adiponectin or leptin level between schistosome-infected and uninfected individuals, nor did we observe any association between adipocytokine levels and BMI.

On the other hand, we observed some differences in serum IFN-γ and IL-18 levels, both of which have been associated with obesity-induced adipose tissue inflammation. Lower levels of these pro-inflammatory adipocytokines could be crucial for explaining why adults with schistosome infection appear to have lower rates of cardiometabolic disease than adults without schistosome infection. Notably, both IFN-γ and IL-18 levels have also been reported to be lower in individuals with *S. japonicum* infection versus healthy controls (9). We conclude, however, that common pathways contributing to obesity in other populations mostly do not explain the differences between BMI and, previously, other measures of adiposity among those with and without schistosome infection in Tanzania (4). Of note, the current study included young Tanzanian adults with an average age of 33.3 years, and only 25% of them were obese or overweight. It is possible that differences could have been detected in older, more obese population (2,10). In addition to this limitation, our analysis was cross-sectional and, therefore, useful for hypothesis generation. Longitudinal data and interventional studies are needed to further test the hypotheses generated by our study.

In conclusion, our study findings highlight a consistent pattern of BMI differences between individuals with *S. mansoni* infection and those without infection, which appears to be correlated with the serum IFN-γ and IL-18 levels, but not with other adipocytokine levels, including leptin, adiponectin, IL-6, TNF-α, and IL-10. Further exploration of the relationship between schistosome infection and BMI may uncover new pathways for targeting obesity using lessons learned from a parasitic infection.

## Data Accessibility Statement

Deidentified data may be available upon request to qualified researchers who meet the criteria for access to confidential data. Interested researchers may contact the corresponding author.
